# Examining the Utility of a Telehealth Warm Handoff in Integrated Primary Care for Improving Patient Engagement in Mental Health Treatment: Randomized Video Vignette Study

**DOI:** 10.2196/40274

**Published:** 2023-06-20

**Authors:** Alex R Fountaine, Megumi M Iyar, Lesley D Lutes

**Affiliations:** 1 The Center for Obesity and Well-Being Research Excellence Department of Psychology University of British Columbia - Okanagan Campus Kelowna, BC Canada

**Keywords:** integrated primary care, integrated care, patient-centered medical home, warm handoff, treatment engagement, collaborative care

## Abstract

**Background:**

A warm handoff from a physician to a mental health provider is often patients’ first contact with psychological services and provides a unique opportunity for improving treatment engagement in integrated primary care (IPC) settings.

**Objective:**

In light of the COVID-19 pandemic, this study sought to examine the impact of different types of telehealth mental health referrals on both the anticipated likelihood of accepting treatment services and anticipated likelihood of continued treatment engagement.

**Methods:**

A convenience sample of young adults (N=560) was randomized to view 1 of 3 video vignettes: warm handoff in IPC, referral as usual (RAU) in IPC, or RAU in standard primary care.

**Results:**

Logistic associations between referral type and the likelihood of referral acceptance (*χ*^2^_1_=10.9, *P*=.004) and the likelihood of continued engagement (*χ*^2^_1_=32.6, *P*<.001) were significant. Participants who received a warm handoff were significantly more likely to anticipate both accepting the referral (b=0.35; *P*=.002; odds ratio 1.42, 95% CI 1.15-1.77) and engaging in continued treatment (b=0.62; *P*<.001; odds ratio 1.87, 95% CI 1.49-2.34) compared with those who received RAU in the standard primary care condition. Furthermore, 77.9% (436/560) of the sample indicated that they would be at least somewhat likely to access IPC mental health services for their own mental health concerns if they were readily available in their own primary care physician’s office.

**Conclusions:**

A telehealth warm handoff resulted in the increased anticipated likelihood of both initial and continued engagement in mental health treatment. A telehealth warm handoff may have utility in fostering the uptake of mental health treatment. Nonetheless, a longitudinal assessment in a primary care clinic of the utility of a warm handoff for fostering referral acceptance and continued treatment engagement is needed to hone the adoptability of a warm handoff process and demonstrate practical evidence of effectiveness. The optimization of a warm handoff would also benefit from additional studies examining patient and provider perspectives about the factors affecting treatment engagement in IPC settings.

## Introduction

### The Existing Need for Integrated Primary Care

An estimated 1.6 million Canadians report unmet mental health care needs each year [1]. Inadequate management of mental health and related concerns can result in consequences including increased severity of psychological symptoms, decreased quality of life, significant individual and systemic economic burden, and decreased life expectancy [1,2]. Although evidence-based psychological services provided by regulated mental health professionals are available in Canada, they are not subsidized by the government and, as such, many individuals, and often those most in need, cannot afford these services [1]. Primary care is often considered the *de facto* source of mental health services in Canada, with studies indicating that up to 80% of Canadians rely on their primary care physician when seeking mental health services [1,2]. However, many of these practitioners have reported lacking sufficient training, resources, and time to meet the demands and scope of mental health concerns currently being presented in primary care [1,2]. In response to the rising need for access to psychological services, integrated primary care (IPC)—the incorporation of mental health services and providers into primary care settings—has increasingly been given unique consideration to meet these demands [3]. Decades of empirical support demonstrates the advantages of IPC models, including reduced wait times, improved accessibility of mental health services, improved mental and physical health outcomes, increased patient and clinician satisfaction, and reduced economic burden of mental health [4-6].

### A Warm Handoff

A feature that is often considered to be integral for fostering patient engagement with psychological services within IPC is a warm handoff. A warm handoff is a type of referral approach in which the primary care physician directly introduces the patient to the psychologist or mental health provider in a face-to-face encounter at the time of the patient’s medical visit [7,8]. Within IPC, most patients’ first point of contact with mental health services and providers is during a “warm handoff” from the primary care physician. Thus, the aim of a warm handoff is to establish a trusting therapeutic alliance between the patient and mental health provider, whereby the patient considers the novel provider to be an integral and trusted member of the health care team [8]. Most of the available literature supports the utility of a warm handoff in engaging patients in treatment with IPC services and providers [9-11]. Moreover, clinicians and experts endorse the use of a warm handoff, noting its utility in practice and from patient feedback about the benefits of a warm handoff including reducing feelings of stigma, providing early access to mental health services, and establishing trusting therapeutic alliances [8,9,12-15].

### The COVID-19 Pandemic and the Rising Importance of Telehealth Care

Although evidence has previously demonstrated that Canadians’ mental health concerns were not being adequately addressed before the pandemic, recent studies suggest that Canadians’ mental health concerns have only been exacerbated by COVID-19 [16-18]. Studies regarding the mental health consequences stemming from COVID-19–related outcomes indicate that the Canadian health care system will be forced to address these sequelae for decades to come [16]. A recent inquiry into the effects of the pandemic on mental health in Canada, spearheaded by the Canadian Mental Health Association and the University of British Columbia, has revealed the preliminary extent of the detrimental mental health impacts of the pandemic [17]. These results demonstrate that the mental health effects of pandemic have been extensive, with approximately 40% of the 3000 respondents indicating that their mental health had deteriorated over the first few months of the pandemic and 46% reporting that they felt anxious and worried [17]. Moreover, the consequences of the pandemic (eg, isolation) have been found to have had great negative effects for those who were already struggling with their mental health. In particular, these individuals (59%) were twice as likely to report that their mental health has declined owing to COVID-19, reporting a 3-fold increase in difficulty with coping and a 4-fold increase in the experience of suicidal thoughts [17,18]. Furthermore, few individuals with existing mental health concerns reported engaging with in-person (2%) or web-based (14%) mental health care during COVID-19, and some of them (5%) were not accessing web-based resources [17].

It is understood that physical distancing associated with the COVID-19 pandemic has been compounding individuals’ existing mental health concerns and inhibiting the help-seeking behaviors of those who are affected [17]. Furthermore, it can be reasonably assumed that these guidelines may also be placing additional hinderance on the capacity for interprofessional communication and successful patient handoffs within IPC settings. According to a 2020 report from the College of Family Physicians of Canada [19], about 80% of patient visits occurred via telehealth (eg, during the pandemic), with many experts questioning whether this modality would become the new normal [20,21]. Experts have offered some recommendations for adapting a warm handoff to a telehealth context, such as placing extra emphasis on instilling patient trust in the new provider as a part of their care team, establishing standard telehealth handoff procedures and scripts, and anticipating patient questions and concerns regarding IPC mental health services [12]. However, to the best of our knowledge, no study has examined the effectiveness of a telehealth warm handoff for improving the likelihood of initial and continued engagement with mental health services. Furthermore, given that a key feature of a warm handoff is the in-person interaction with both the primary care physician and the mental health provider, it will be important to assess the utility of a warm handoff within a telehealth context.

This study sought to examine whether a telehealth warm handoff to an IPC mental health professional increases the likelihood of initial acceptance of the referral and intent to continue treatment, compared with the *referral as usual* (RAU) process, used in both IPC and standard primary care. To answer this question, vignettes were applied. It was hypothesized that a telehealth warm handoff would promote both great initial acceptance of the referral and great intent to continue treatment, relative to an RAU in a standard primary care setting. Furthermore, we conducted moderation analyses to control for the influence of participants’ own current mental and physical health status on their responses to the 2 treatment engagement variables.

## Methods

### Study Design

The study used a 3-arm between-participants video vignette design and was conducted between March 2021 and April 2021. Participants were students enrolled in an undergraduate degree and were randomized into one of three vignette conditions: (1) warm handoff in an IPC context; (2) RAU in IPC; or (3) RAU in standard primary care, in which they received a hypothetical telehealth referral from a primary care physician and then indicated both their likelihood of referral acceptance and of continuing treatment.

### Recruitment and Eligibility

Student participants were recruited through Sona Systems, the psychology department’s portal for web-based study recruitment. All participants were asked to complete various web-based questionnaires, which were available to be completed confidentially through a web-based survey hosted on Qualtrics [22]. Eligibility criteria required participants to be aged ≥18 years; be fluent in English; and be a Canadian citizen, be a permanent resident of Canada, or have been residing in Canada for at least 12 months before completing the study.

### Ethics Approval, Informed Consent, and Participation

This study was conducted in accordance with the Tri-Council Policy Statement–2: Ethical Conduct for Research Involving Humans and received approval from the University of British Columbia Okanagan Behavioral Research Ethics Board before conducting any research activities (H20-03130-A001). Each participant was automatically assigned an anonymous participant ID by Qualtrics (ie, data were deidentified). Thus, no participant could be identified at any point in our research records and outputs. The consent form stated that participation in the study was completely voluntary, and participants were able to withdraw at any point without penalty. As remuneration, participants received course credit (ie, 0.5 credits per 0.5 hours of participation).

### Measures and Stimuli

#### Vignettes

The vignettes consisted of two components: (1) a brief written summary of their hypothetical visit and (2) a short video, wherein a mock physician performed 1 of the 3 types of telehealth referrals depending on the participant’s experimental condition. Participants were first asked to imagine that they were a patient attending a telehealth appointment hosted on the teleconferencing platform, Zoom (version 5.4.7; Zoom Video Communications, Inc), with their primary care physician. They then carefully read the brief summary of their appointment with their physician. The summary informed the participant about the relevant contextual factors of this visit, including their physical and mental health history, their mental health and related concerns over the past 2 months, and the context in which their visit occurred (either in an IPC clinic or a standard primary care clinic). The 3 video vignettes varied in terms of the type of referral they received and in what context they received the referral, resulting in the three experimental conditions (ie, types of referrals): (1) a warm handoff in IPC, (2) an RAU in IPC, and (3) an RAU in standard primary care. RAU was operationalized as a prescribed referral to other provider services at a later time [11]. In the RAU in IPC condition, participants were referred to the same provider as in the warm handoff condition; however, instead of being introduced to the mental health service provider at the time of the initial visit, they were simply scheduled for a subsequent appointment with the provider. For an RAU in standard primary care, participants were simply referred to an outpatient mental health service.

Given the importance of ensuring that a warm handoff used within the corresponding vignette reflected a genuine, real-world account of what may occur in an IPC clinic, a comprehensive sample of resources including reputable YouTube videos, peer-reviewed journal articles, and integrated health care organization sample scripts and web pages were referred to [8,23-27]. To assess the face validity of the warm handoff vignette, a group of behavioral health consultants and supervising registered clinical psychologists working in an integrated behavioral medicine program operating in Canada were consulted in the vignette development phase of this study. For their consultative role, the behavioral health consultants and supervising psychologists met with the main study team to review the scripts, offer their insights, and provide recommendations to improve applicability of the warm handoff.

#### Treatment Engagement Outcomes

This study had two a priori outcomes of behavioral intent to engage with treatment, in which participants were asked to rate their likelihood of (1) accepting the referral and (2) continuing to engage with the mental health service to which they were referred on a 5-point Likert scale, ranging from 1 (very unlikely) to 5 (very likely).

#### Demographic Questionnaire

Participants were asked to complete a demographic questionnaire that included measures of age; ethnicity; nationality; sex; whether they have a primary care physician; and how well their mental health care needs have been met within the past year, measured on a 5-point Likert scale, ranging from 1 (completely unmet) to 5 (completely met). Following the vignette, all participants were then asked to read the same passage, which provided a brief description of IPC; they were then asked to rate how likely they would be to access IPC services for their own mental health concerns if these services were readily available within their primary care physicians’ office. This was measured on a 5-point Likert scale, ranging from 1 (very unlikely) to 5 (very likely).

#### Measures of Psychological and Physical Health

Measures of psychological health included the Generalized Anxiety Disorder scale–7, used to measure generalized anxiety symptoms [28]; Patient Health Questionnaire–9, used to measure depressed mood [29]; and Satisfaction With Life Scale, used to measure life satisfaction—a facet of subjective well-being [30]. Selected items from the Fixed Core sections of the Brief Risk Factor Surveillance System Questionnaire [31] were used to assess various components of an individual’s physical health status. Specifically, participants were asked to indicate the number of days in the past month in which (1) their physical health was not good and (2) they were unable to do their usual activities owing to poor physical health. In addition, participants were asked to rate their health in general from 1 (excellent) to 5 (poor). These 3 items have been previously used to measure health status [32].

### Procedure

Following the provision of informed consent, participants followed the instructions provided and completed the web-based measures. Participants were randomly assigned to 1 of 3 experimental conditions, in which they were asked to imagine that they were a patient who was having a telehealth checkup appointment with their primary care physician. Next, each participant was asked to carefully read the brief summary of their appointment with their physician. Participants were then asked to carefully view a video, in which the physician engaged in either a warm handoff or an RAU, depending on the experimental condition they were randomly assigned to. Next, they rated both their likelihood of accepting the initial referral and their likelihood of engaging in continued treatment with the mental health service to which they were referred. Following the vignette, all participants read the brief description of IPC and rated how likely they would be to access IPC services for their own mental health concerns if these services were readily available within their primary care physicians’ office. Participants then completed the previously mentioned demographic questionnaire and were provided with a debriefing form, including a list of relevant mental health resources.

### Analytical Methods

Descriptive statistics were calculated for demographic variables. Logistic regressions were conducted as omnibus tests of associations between the type of referral received and the 2 treatment engagement outcomes, comparing both a warm handoff and RAU in IPC conditions against the control group (RAU in standard primary care). Moderated logistic regressions were then used to control for the influence of participants’ own mental health status on their responses to the 2 engagement outcomes as the individual in the vignette. Observed effects for these null hypothesis significance testing analyses were deemed to be significant for *P*<.05. An a priori power calculation was conducted in G*Power using a recommended small effect size (odds ratio=1.5), Cronbach α=.05, and 1 − β=.95, which indicated that a final sample of 337 would be needed to detect a statistically significant effect, should one exist [33,34]. All analyses were conducted using RStudio (version 1.2.1335; RStudio Team) [35].

## Results

### Overview

A total of 560 undergraduate participants registered for and completed the study. The sample was predominantly female (402/560, 71.8%) and White (326/560, 58.2%), with average age of 20.3 (SD 3.8) years. Results showed there were no significant differences among groups regarding demographic characteristics (all *P*<.05; Table 1). Participants reported moderate levels of depression and anxiety on average (Table 1). Furthermore, 24.5% (137/560) of the participants indicated that they did not currently have a primary care physician. On average, participants reported that their mental health care needs were somewhat met (mean 3.26, SD 1.13) during the past year. However, a substantial portion of the sample (254/560, 45.4%) indicated that their mental health care needs were not being adequately met (ie, indicated that their mental health care needs were either somewhat or completely unmet). Furthermore, on average, participants indicated that they would be somewhat likely to access IPC mental health services for their own mental health concerns if they were available within their primary care physicians’ office (median 4, SD 0.86), with approximately 77.9% (436/560) of the sample indicating that they would be at least somewhat likely to access these services.

**Table 1:** Sample demographic characteristics.

Characteristics		Overall (N=560)	WH^a^ (n=188)	RAU-I^b^ (n=186)	RAU-S^c^ (n=186)	*χ*^2^ (*df*)^d^	*P* value^d^
**Sex, n (%)**						4.1 (4)	.49
	Female	402 (71.8)	132 (70.2)	137 (73.7)	133 (71.5)		
	Male	150 (26.8)	55 (29.3)	45 (24.2)	50 (26.9)		
	Other	1 (0.2)	1 (0.5)	2 (1.1)	1 (0.5)		
	N/A^e^	2 (0.4)	0 (0)	2 (1.1)	0 (0)		
**Ethnicity, n (%)**						7.8 (14)	.92
	African	15 (2.7)	5 (2.7)	4 (2.2)	6 (3.2)		
	Asian	115 (20.5)	40 (21.3)	34 (18.3)	41 (22)		
	East Indian	31 (5.5)	10 (5.3)	11 (5.9)	10 (5.4)		
	Hispanic	13 (2.3)	6 (3.2)	2 (1.1)	5 (2.7)		
	Indigenous	18 (3.2)	4 (2.1)	6 (3.2)	8 (4.3)		
	Middle Eastern	15 (2.7)	3 (1.6)	8 (4.3)	4 (2.2)		
	White	326 (58.2)	111 (59)	112 (60.2)	103 (55.4)		
	Other	27 (4.8)	9 (4.8)	9 (4.8)	9 (4.8)		
Age (years), mean (SD)		20.6 (3.89)	20.5 (2.89)	20.7 (3.81)	20.6 (4.77)	7.7 (8)	.19
PHQ-9^f^ total, mean (SD)		10.1 (6.61)	10.7 (6.50)	10.4 (7)	9.14 (6.22)	7.3 (10)	.11
GAD-7^g^ total, mean (SD)		9.4 (5.56)	9.8 (5.58)	9.8 (5.68)	8.6 (5.36)	4.8 (9)	.57
SWLS^h^ total, mean (SD)		21.9 (6.12)	21 (6.28)	22.2 (5.99)	22.4 (6.01)	5.4 (12)	.72
BRFSS (health status)^i^, mean (SD)		2.5 (0.94)	2.5 (0.99)	2.4 (0.92)	2.4 (0.92)	9.5 (8)	.30
BRFSS (sick days)^i^, mean (SD)		7.5 (8.02)	7.51 (7.75)	7.83 (8.25)	7.05 (8.08)	8.1 (8)	.86

^a^WH: warm handoff in integrated primary care.

^b^RAU-I: referral as usual in integrated primary care.

^c^RAU-S: referral as usual in standard primary care.

^d^Chi-square test for differences between groups with associated *P* values.

^e^N/A: not applicable.

^f^PHQ-9: Patient Health Questionnaire–9—used to measure depressed mood on a Likert scale ranging from 0 (not at all) to 3 (nearly every day), with high scores indicating great severity.

^g^GAD-7: Generalized Anxiety Disorder scale–7—used to measure anxiety symptoms from 0 (not at all) to 3 (nearly every day), with high scores indicating great symptom severity.

^h^SWLS: 5-item Satisfaction With Life Scale—measured from 1 (strongly disagree) to 7 (strongly agree), where high scores indicate great life satisfaction.

^i^BRFSS: Brief Risk Factor Surveillance Survey—selected questions were used to quantify participants’ physical health (ie, health status and sick days).

### Primary Engagement Outcomes

#### Likelihood of Referral Acceptance

Most participants (441/560, 78.8%) indicated that they would accept an initial referral regardless of the type of referral they received. Correspondingly, the logistic regression analysis revealed a significant association between the type of referral received and the likelihood of initial referral acceptance (*χ*^2^_1_=10.9, *P*=.004), whereby those who received a telehealth warm handoff were significantly more likely to accept the referral compared with those who received an RAU in the standard primary care context (*P*=.02; Table 2). In contrast, those who received an RAU in IPC were not significantly more likely to accept the referral than those who received an RAU in standard primary care (*P*=.33).

**Table 2:** Simple logistic associations between referral type and engagement outcomes (N=560).

			b (SE)		*P* value		OR^a^ (95% CI)		LLR^b^			*χ*^2^ (*df*)			*P* value	
**Model 1—referral acceptance**										−609.76			10.9 (1)			.004
	Warm handoff	0.35 (0.11)		.02		1.43 (1.14-1.77)										
	RAU-I^c^	0.10 (0.10)		.33		1.11 (0.90-1.35)										
**Model 2—continued engagement**										−598.93			32.6 (1)			.001
	Warm handoff	0.62 (0.11)		.001		1.87 (1.49-2.34)										
	RAU-I	0.30 (0.10)		.005		1.34 (1.10-1.65)										

^a^OR: odds ratio.

^b^LLR: log likelihood ratio.

^c^RAU-I: referral as usual in integrated primary care.

#### Likelihood of Continued Treatment Engagement

Approximately two-thirds of the sample (363/560, 64.8%) indicated that they would be least somewhat likely to engage in continued treatment, regardless of the referral received. Accordingly, the logistic regression analysis also revealed a significant association between the type of referral and the likelihood of engaging in continued treatment (*P*=.001). Specifically, those who received either a warm handoff (*χ*^2^_1_=32,6, *P*=.001) or an RAU in IPC (*P*=.005) were significantly more likely to continue to engage in treatment with the service and provider to which they were referred, compared with those who received RAU in the standard primary care context (Table 2).

### Controlling for Influence of Participant Characteristics on Responses

#### Likelihood of Referral Acceptance

After examining the moderating influence of participants’ mental and physical health status on the relationship between referral type and the likelihood of referral acceptance, the association was still significant (*χ*^2^_1_=23.3, *P*=.048; Table 3). Those who received a warm handoff were significantly more likely to accept the referral, compared with those who received an RAU in a standard primary care context (*P*=.002). Relatedly, participants’ mental and physical health status did not have significant individual or combined influence on the likelihood of acceptance (all *P*>.05). The inclusion of mental and physical health status explains significant additional variance in our observation.

**Table 3:** Moderated logistic associations between referral type and referral acceptance (N=560).

Model 3—moderation^a,b,c^		b (SE)	*P* value	OR^d^ (95% CI)
**Warm handoff**		0.36 (0.12)	.002	1.43 (1.14-1.80)
	PHQ-9^e^	0.05 (0.04)	.26	1.05 (0.97-1.14)
	GAD-7^f^	0.04 (0.04)	.30	1.05 (0.96-1.13)
	BRFSS^g^—health status	0.09 (0.18)	.61	1.09 (0.77-1.53)
	BRFSS—sick days	0 (0.04)	.98	1 (0.92-1.09)
	PHQ-9 × GAD-7	−0.02 (0)	.58	1 (0.97-1.14)
	Health status × sick days	−0.07 (0.01)	.61	1 (0.77-1.54)
**RAU-I^h^**		0.10 (0.11)	.37	1.10 (0.89-1.40)
	PHQ-9	−0.03 (0.04)	.52	0.97 (0.90-1.06)
	GAD-7	0.05 (0.04)	.90	1.01 (0.93-1.09)
	BRFSS—health status	0.04 (0.18)	.79	1.05 (0.74-1.49)
	BRFSS—sick days	0.04 (0.04)	.36	1.04 (0.96-1.13)
	PHQ-9 × GAD-7	0 (0)	.33	1 (0.90-1.06)
	Health status × sick days	−0.01 (0.01)	.33	0.99 (0.77-1.54)

^a^Log likelihood ratio=−593.62.

^b^*χ*^2^_1_=23.3.

^c^*P*=.048.

^d^OR: odds ratio.

^e^PHQ-9: Patient Health Questionnaire–9—used to measure depressed mood on a Likert scale ranging from 0 (not at all) to 3 (nearly every day), with high scores indicating high severity.

^f^GAD-7: Generalized Anxiety Disorder scale–7—used to measure anxiety on a Likert scale ranging from 0 (not at all) to 3 (nearly every day), with high scores indicating high symptom severity.

^g^BRFSS: Brief Risk Factor Surveillance Survey—selected questions were used to quantify participants’ physical health (ie, health status and sick days).

^h^RAU-I: referral as usual in integrated primary care.

#### Likelihood of Continued Treatment Engagement

Similarly, after examining the moderating influence of participants’ Patient Health Questionnaire–9 and Generalized Anxiety Disorder scale–7 scores, the association between referral type and likelihood of continued engagement in treatment was significant (*χ*^2^_1_=46.4, *P*=.001; Table 4). Those who received either a warm handoff (*P*=.001) or an RAU in IPC (*P*=.005) were significantly more likely to continue to engage, compared with those who received an RAU in a standard primary care context. Again, participants’ mental and physical health markers did not have significant individual or combined influence on the likelihood of continued treatment engagement and explained significant additional variance (all *P*>.05).

**Table 4:** Moderated logistic associations between referral type and continued engagement (N=560).

Model 4—moderation^a,b,c^			B (SE)		*P* value		OR^d^ (95% CI)
**Warm handoff**			0.65 (0.12)		.001		1.92 (1.52-2.42)
	PHQ-9^e^	0.06 (0.04)		.18		1.06 (0.97-1.15)	
	GAD-7^f^	0.04 (0.04)		.41		1.04 (0.95-1.13)	
	BRFSS^g^—health status	0.13 (0.18)		.47		1.14 (0.80-1.62)	
	BRFSS—sick days	−0.01 (0.04)		.91		0.99 (0.91-1.09)	
	PHQ-9 × GAD-7	0 (0)		.56		1 (0.97-1.15)	
	Health status × sick days	−0.01 (0.01)		.63		0.99 (0.80-1.62)	
**RAU–Integrated^h^**			0.30 (0.12)		.005		1.35 (1.09-1.66)
	PHQ-9	−0.02 (0.04)		.57		0.98 (0.90-1.06)	
	GAD-7	0 (0.04)		.99		1 (0.92-1.09)	
	BRFSS—health status	0.07 (0.18)		.70		1.07 (0.75-1.53)	
	BRFSS—sick days	0.04 (0.04)		.40		1.04 (0.95-1.13)	
	PHQ-9 × GAD-7	0 (0)		.32		1 (0.90-1.06)	
	Health status × sick days	−0.01 (0.01)		.33		0.99 (0.75-1.53)	

^a^Log likelihood ratio=−582.1.

^b^*χ*^2^_1_=46.4.

^c^*P*=.001.

^d^OR: odds ratio.

^e^PHQ-9: Patient Health Questionnaire–9—a 9-item self-report depression screening instrument—used to measure depressed mood over the past 2 weeks on a Likert scale ranging from 0 (not at all) to 3 (nearly every day), with high scores indicating high severity.

^f^GAD-7: Generalized Anxiety Disorder scale–7—a 7-item self-report anxiety screening questionnaire—used to measure generalized anxiety symptoms on a Likert scale ranging from 0 (not at all) to 3 (nearly every day), with high scores indicating high symptom severity.

^g^BRFSS: Brief Risk Factor Surveillance Survey—selected items were used to quantify participants’ physical health (ie, health status and sick days).

^h^RAU-I: referral as usual in integrated primary care.

## Discussion

### Principal Findings

Given the substantial transition to telehealth care prompted by the pandemic, coupled with the recent increase in mental health needs, studies are needed to examine the best ways to provide mental health services and engage patients in treatment in this new modality of health care delivery. The purpose of this study was to examine the utility of a warm handoff for improving the anticipated likelihood of patient engagement in mental health treatment in primary care, as compared with that of the standard referral approach, used in both integrated and standard primary care contexts. As hypothesized, participants who received a telehealth warm handoff were significantly more likely to anticipate accepting the mental health referral than those individuals who received an RAU in a standard primary care setting. Notably, a warm handoff was the only significant individual predictor. This suggests that the overall association between the type of referral received and the likelihood of accepting the referral was driven by the difference between a warm handoff and the RAU in standard primary care conditions. Moreover, this positive association between the type of referral received and the likelihood of accepting the referral was actually strengthened after controlling for participants’ mental and physical health status. Similarly, the association between the type of referral received and the anticipated likelihood of continuing to engage with the service and provider to which they were referred was also significant. Specifically, participants who received either a warm handoff or an RAU in IPC were significantly more likely to anticipate continuing to engage in treatment, compared with those who received an RAU in standard primary care. The association between the type of referral received and the anticipated likelihood of continuing to engage in treatment was strengthened after controlling for participants’ mental and physical health status.

Our results are consistent with the literature regarding the efficacy of in-person warm handoff for engaging patients in mental health treatment. In a study using a retrospective cohort design, patients who were referred using a warm handoff were found to have low nonattendance rates, increased mental health service engagement, and decreased wait times for appointment compared with those who were “referred as usual” [11]. These results suggest that the mere presence of mental health services in the primary care setting may not be sufficient to engage patients; it is the level of integration and quality of care being provided (eg, the use of warm handoffs) that truly matters for engaging patients in initial and continued mental health treatment. Furthermore, the personal introduction to the new provider, which is provided by a warm handoff, may meaningfully reduce the stigma and hesitancy to engage in mental health treatment. Previous studies found that individuals who receive mental health care in IPC contexts have reported great trust in their primary care provider compared with those in standard, nonintegrated contexts, whereby they may be more likely to continuously engage with a provider who is known to and trusted by their physician [9].

An important area of findings that were particularly noteworthy was related to the large proportion of individuals, regardless of their study arm allocation, who anticipated being likely to accept the initial referral to the mental health service to which they were referred; this result was mirrored in participants’ personal anticipated likelihood to engage with mental health services if they were readily available in their own primary care physicians’ office (ie, not hypothetical). Specifically, approximately 80% (441/560, 78.8%) of the total sample indicated that they anticipate being at least somewhat likely to accept an initial referral, and 64.8% (363/560) would be at least somewhat likely to engage in continued treatment. Furthermore, most of the sample (436/560, 77.9%) indicated that they personally would be likely to engage with mental health services for their own mental health concerns if they were readily available in their own primary care physician’s office (ie, IPC), and none of the individuals indicated that they would be very unlikely to access these services. Such results are incredibly promising—especially when considering the moderate levels of anxiety and depression and unmet mental health care needs reported by the participants. This indicates that, if mental health providers were integrated into primary care, a sizable portion of this population anticipate being at least somewhat likely to accept a telehealth mental health referral for their mental health related concerns. Previous studies identify not knowing where or how to access services as one of the largest barriers to mental health service use reported by young adults [36-38]. Intriguingly, a recent study indicated that among those with mental health related concerns during the time of COVID-19, only 14% are accessing telehealth mental health services [17]. However, the results from this study suggest that the reason that individuals are not accessing mental health services may not be owing to the lack of need but rather owing to the lack of accessibility to evidence-based mental health care provided or supervised by highly trained, regulated mental health providers. Decades of empirical evidence suggests that IPC models lead to reduced wait times for mental health and medical services, improved access to mental health and medical services, improved physical and mental health, improved patient and clinician satisfaction, and reduced economic burden of mental health [4-6]. Therefore, in consideration of previous evidence—coupled with the results of this study—IPC models appear to provide a unique and viable opportunity to adequately address the increasing scope and severity of Canadians’ current mental health concerns.

### Limitations

Although this study has many strengths, there are several limitations that should be discussed. First and most notably, this study measured behavioral intent for the initial and continued uptake of services in a hypothetical vignette, not the actual uptake of services (eg, attendance at a subsequent appointment). However, a growing body of literature has demonstrated that behavioral intent and perception that the proposed intervention will be effective are key contributing factors to an individual’s actual likelihood of continuously engaging with treatment [37-41]. Moreover, when participants were asked about their own personal likelihood to engage in initial and continued treatment in IPC, responses were consistent with a hypothetical warm handoff in IPC condition. Consequently, these observed results provide information regarding the current demand for and likelihood of uptake of mental health services among this high-risk population [16-18]. Nonetheless, a longitudinal assessment in a primary care clinic of the utility of a telehealth warm handoff for fostering referral acceptance and continued treatment engagement is needed to hone the adoptability of a telehealth warm handoff process and demonstrate practical evidence of effectiveness.

Relatedly, another limitation was that the current sample was a convenience sample of undergraduate student population, which has previously been identified in the literature as posing a potential limitation to the generalizability of study results [42]. However, despite not being a recruitment criterion, the current sample reported moderate levels of depression and anxiety on average, with <50% of the sample reporting that their mental health needs were being adequately met. As such, it is evident that participants who were sampled in this study represent a key vulnerable population—young adults who are already struggling with their mental health and not receiving adequate care [1]. Notably, the same issues identified in our sample—unmet mental health needs, current psychological distress, and the lack of a primary care physician—are also currently being reported among the general community at a similar rate [16-18,43,44]. Moreover, although future studies should certainly examine the utility of a telehealth warm handoff among a community sample, this study evidently examined a critically vulnerable population, the importance of which should not be understated. Finally, certain moderating variables including the patient’s level of trust in their physician [13,45,46], their attitude toward mental illness and help seeking [36,47,48], and patient-physician race concordance were not measured [39]. Future studies should consider including these variables to improve our understanding of factors that influence the acceptance of different types of telehealth mental health referrals and continued treatment engagement within IPC contexts, wherein the nature of these phenomena is not well documented. In addition, future studies should continue to focus on elucidating the components of a warm handoff that are deemed to be most appreciated by or beneficial to patients, allowing for the optimization of the warm handoff process, in both in-person and telehealth contexts.

### Conclusions

These results suggest that the use of a telehealth warm handoff may foster young adults’ likelihood of engaging with mental health services in primary care, thereby providing increased access to the mental health care that they require. Furthermore, our results suggest that the telehealth modality of health care delivery does not appear to be a substantial potential barrier to individuals’ willingness to receive mental health services, as evidenced by the large proportion of the sample that anticipate being likely to accept the referral and continuing engagement with the treatment. This has important implications, given that telehealth IPC services have the potential to provide patients from rural and remote communities better access to efficacious psychological services, minimizing yet another barrier to accessing evidence-based mental health care provided or supervised by highly trained, regulated mental health providers. Moreover, our findings indicate that a considerable proportion of young adults are experiencing substantial mental health concerns and that these mental health concerns have generally not been addressed sufficiently. However, our findings also indicate that young adults are generally very willing to accept and engage with mental health services in an IPC setting. Such a result suggests that implementing IPC models on a widespread scale would likely be a highly endorsed solution for addressing the “shadow pandemic of mental health issues” [49] and for providing young adults with great access to imminently needed mental health care. We urge researchers to continue examining the practical considerations for implementing warm handoff in a telehealth context and policy makers and stakeholders to seriously consider investing in the integration of mental health care as a core component of primary care health services in Canada.

## Appendix

Multimedia Appendix 1 Data file. Cleaned data file that was used when conducting all statistical analyses from which conclusions were drawn.

## Abbreviations

IPC: integrated primary care
RAU: referral as usual

## Data Availability

All data generated or analyzed during this study are included in this published paper (refer to Multimedia Appendix 1).
